# Annual Incidence of Hospitalization for Nonfatal Firearm-Related Injuries in New York From 2005 to 2016

**DOI:** 10.1001/jamanetworkopen.2021.15713

**Published:** 2021-07-28

**Authors:** Yu-Tien Hsu, Ya-Wen Chen, David C. Chang, Numa P. Perez, Maggie L. Westfal, Ya-Ching Hung, Cassandra M. Kelleher, Peter T. Masiakos, Chana A. Sacks

**Affiliations:** 1 Department of Surgery, Massachusetts General Hospital, Harvard Medical School, Boston; 2 Division of General Internal Medicine and Mongan Institute, Department of Medicine, Massachusetts General Hospital, Harvard Medical School, Boston

## Abstract

**Importance:**

In the US, approximately 40 000 people die due to firearm-related injuries annually. However, nonfatal firearm-related injuries are less precisely tracked.

**Objectives:**

To assess the annual incidence of hospitalization for nonfatal firearm-related injuries in New York and to compare the annual incidence by sex, race/ethnicity, county of residence, and calendar years.

**Design, Setting, and Participants:**

This retrospective cross-sectional study used data from the New York Statewide Planning and Research Cooperative System for patients aged 15 years or older who presented to an emergency department in New York with nonfatal firearm-related injuries from January 1, 2005, to December 31, 2016. Data were analyzed from January 15, 2019, to April 21, 2021.

**Exposure:**

A nonfatal firearm-related injury, defined by *International Classification of Diseases, Ninth Revision, Clinical Modification* and *International Statistical Classification of Diseases, Tenth Revision, Clinical Modification* codes.

**Main Outcomes and Measures:**

The annual incidence of nonfatal firearm-related injuries was calculated by determining the number of patients with a nonfatal firearm-related injury each year divided by the total population of New York.

**Results:**

The study included 31 060 unique patients with 35 059 hospital encounters for nonfatal firearm-related injuries. The mean (SD) age at admission was 28.5 (11.9) years; most patients were male (90.6%) and non-Hispanic Black individuals (62.0%). The overall annual incidence was 18.4 per 100 000 population. Although decreasing trends of annual incidence were observed across the state during the study period, this trend was not present in all 62 counties, with 32 counties (51.6%) having an increase in the incidence of injuries between 2005 and 2010 and 29 (46.8%) having an increase in the incidence of injuries between 2010 and 2015. In 19 of the 30 counties (63.3%) that had a decrease in incidence in earlier years, the incidence increased in later years.

**Conclusions and Relevance:**

The annual incidence of hospitalization for nonfatal firearm-related injuries in New York during the study period was 18.4 per 100 000 population. Reliable tracking of nonfatal firearm-related injury data may be useful for policy makers, hospital systems, community organizers, and public health officials as they consider resource allocation for trauma systems and injury prevention programs.

## Key Points

**Question:**

What was the annual incidence of hospitalization for nonfatal firearm-related injuries in New York from 2005 to 2016?

**Findings:**

In this cross-sectional study of 31 060 patients with 35 059 hospital encounters for nonfatal firearm-related injuries in New York from 2005 to 2016, the overall annual incidence of hospitalization for nonfatal firearm-related injuries was 18.4 per 100 000 population. The annual incidence of these injuries in the state decreased during the study period, but this trend was not observed in all counties.

**Meaning:**

These findings may be useful for policy makers and public health officials as they consider resource allocation for trauma systems and injury prevention programs.

## Teaser

This cross-sectional study uses data from the New York Statewide Planning and Research Cooperative System to assess the annual incidence of hospitalization for nonfatal firearm-related injuries in New York overall and by sex, race/ethnicity, county of residence, and calendar year from 2005 to 2016.

## Introduction

In the US, approximately 40 000 people die due to firearm-related injuries each year.^1^ This is similar to the number of deaths that occur each year from motor vehicle crashes or liver disease.^2^ However, compared with other leading causes of death, firearm-related violence remains underrepresented in the scientific literature in part owing to the paucity of extramural funding. One study suggested that this area of investigation receives 1.6% of expected research funding compared with other leading causes of death.^3^

This lack of investment is apparent in the absence of infrastructure to reliably track nonfatal firearm-related injuries. Although deaths are publicly reported and reliably described, national data on nonfatal firearm-related injuries are limited.^4^ For example, the US Centers for Disease Control and Prevention (CDC) reported that 133 895 (95% CI, 31 329-236 461) nonfatal firearm-related injuries occurred in 2017^5^—an estimate so imprecise as to render it unhelpful in understanding the scope of this problem. This lack of precision was the result of using a small number of hospitals to derive the estimates, which have been increasingly imprecise over time.^6^ More recently, the CDC deemed the nonfatal firearm-related injury data so unreliable that the estimates are no longer publicly available.^5^ Without reliable data, it is challenging to understand the scope of the problem, to develop prevention strategies, and to track their effectiveness.

Although federal data on nonfatal firearm-related injuries are unavailable or insufficient, data from state or regional health care systems can provide opportunities to better measure and track these injuries. For example, the New York Statewide Planning and Research Cooperative System (SPARCS) is an all-payer, state-level database that captures 100% of inpatient hospitalizations and emergency department visits within the state.^7^ Such a comprehensive data system offers the ability to examine the county-level incidence of nonfatal firearm-related injuries over time and to explore meaningful covariates associated with geographic differences and other disparities.

This study used New York SPARCS to assess the feasibility of using such a state-level database to evaluate the annual incidence of nonfatal firearm-related injuries in New York. We also compared the annual incidence of nonfatal firearm-related injuries by sex, race/ethnicity, county of residence, and calendar years to examine whether disparities existed in nonfatal firearm-related injuries and their temporal changes. We explored whether differences occurred by geographic location and assessed the association of socioeconomic status with county-level disparities in nonfatal firearm-related injuries.

## Methods

### Database and Cohort Creation

We performed a cross-sectional study using retrospective data obtained from the New York SPARCS database from January 1, 2005, to December 31, 2016, to develop a comprehensive statewide cohort of individuals aged 15 years or older who visited the emergency department for nonfatal firearm-related injuries. The SPARCS database captures 100% of inpatient hospitalizations and emergency department visits throughout the state.^7^ We chose the age cutoff of 15 years because youths aged 15 to 18 years have been shown to have patterns of firearm-related injuries similar to those of the adult population.^8^ This study was in compliance with ethical standards and was approved by the institutional review board committee of the New York State Department of Health. Because all data obtained from SPARCS were deidentified, informed consent was not required. This study followed the Strengthening the Reporting of Observational Studies in Epidemiology (STROBE) reporting guideline for cross-sectional studies.

Firearm-related injuries were identified using *International Classification of Diseases, Ninth Revision, Clinical Modification* (*ICD-9-CM*) external cause of injury codes for patients with a discharge date between January 1, 2005, and September 30, 2015. *International Classification of Diseases, Tenth Revision, Clinical Modification* (*ICD-10-CM*) codes were used for those who were discharged between October 1, 2015, and December 31, 2016. The list of *ICD-9-CM* and *ICD-10-CM* codes used in this study followed those indicated in reports published by the CDC^9,10^ (eTable in the Supplement). Patients who visited an emergency department for firearm-related injuries and survived to hospital discharge were included. Patients who died in the emergency department or during the index hospitalization were excluded.

### Outcomes and Study Variables

The primary outcome was the annual incidence of nonfatal firearm-related injuries, calculated as the number of patients with a nonfatal firearm-related injury each year divided by the total population of New York obtained from the US Census Bureau.^11,12,13^ We compared the annual incidence by age, sex, and race/ethnicity. Race/ethnicity was self-reported and was categorized as non-Hispanic White, non-Hispanic Black, Hispanic, Asian, or other. “Other” included American Indian and Alaska Native, Native Hawaiian and Other Pacific Islander, and 2 or more races.

### Statistical Analysis

Data were analyzed from January 15, 2019, to April 21, 2021. For the descriptive analysis of the demographic and clinical characteristics, we calculated medians (with interquartile ranges) for continuous variables and counts (with percentages) for categorical variables. We calculated the annual incidence of nonfatal firearm-related injuries for each calendar year from 2005 to 2016 and further stratified by sex, race/ethnicity, and county of residence.

We explored the correlation between the county-level annual incidence of nonfatal firearm-related injuries and the county-level median household income from 2010 to 2016 by plotting the incidence of nonfatal firearm-related injuries in a given county and that county’s median income each year and calculating the regression coefficient with 95% CI and *P* value. All dollars were inflation adjusted to 2016 dollars.^14^ The median household income analysis included 39 counties (62.9% of all New York counties) because the US Census Bureau only reported annual estimates of median household income for counties with populations of more than 65 000. We also performed a linear regression to examine whether median household income in 2010 could estimate the change in annual incidence between 2010 and 2015. Temporal changes in annual incidence of nonfatal firearm-related injuries between 2 years were evaluated using 2-proportion *Z* tests.

The threshold of statistical significance was set at α = .05 using 2-sided testing for all analyses. Analyses were performed using Stata, version 15.0 (StataCorp LLC). Heatmaps of the annual incidence and the changes in numbers of nonfatal firearm-related injuries by New York county in specific years were created using Tableau Desktop, version 2020.2 (Tableau Software Inc).

## Results

A total of 35 059 encounters for nonfatal firearm-related injuries sustained by 31 060 unique individuals were assessed (Table). The mean annual number of encounters was 2922 (129.2). Most patients were male (90.6%) and non-Hispanic Black individuals (62.0%). The mean (SD) age at admission was 28.5 (11.9) years. Overall, the median length of stay was 1 day (interquartile range, 0-4.0 days) (with those who were discharged from the emergency department assigned a length of stay of 0 days). Among those admitted to the hospital, the median length of stay was 4.0 days (interquartile range, 2.0-8.0 days). Most encounters (85.0%) were for patients from large metropolitan areas, and 10.7% of patients sustained nonfatal firearm-related injuries more than once during the study period.

**Table.** Baseline Characteristics of Patients With Nonfatal Firearm-Related Injuries in New York From 2005 to 2016

Characteristic	Patients
**Patient-level characteristics (n = 31 060)**	
Sex, No. (%)	
Male	28 152 (90.6)
Female	2904 (9.3)
Missing	4 (0.01)
Race/ethnicity, No. (%)	
Non-Hispanic Black	19 264 (62.0)
Non-Hispanic White	4169 (13.4)
Hispanic	3944 (12.7)
Asian	271 (0.9)
Other^a^	1732 (5.6)
Missing	1680 (5.4)
Hospital encounters per patient, No. (%)	
1	27 723 (89.3)
>1	3337 (10.7)
**Encounter-level characteristics (n = 35 059)**	
Age, mean (SD), y	28.5 (11.9)
Length of stay, median (IQR), d	1 (0-4)
Urbanicity of the county of residence, No. (%)	
Large metropolitan	29 807 (85.0)
Medium metropolitan	2760 (7.9)
Small metropolitan	639 (1.8)
Micropolitan	634 (1.8)
Noncore	318 (0.9)
Missing	901 (2.6)

Abbreviation: IQR, interquartile range.

^a^ Other includes American Indian and Alaska Native, Native Hawaiian and other Pacific Islander, and 2 or more races.

**Figure 1.**
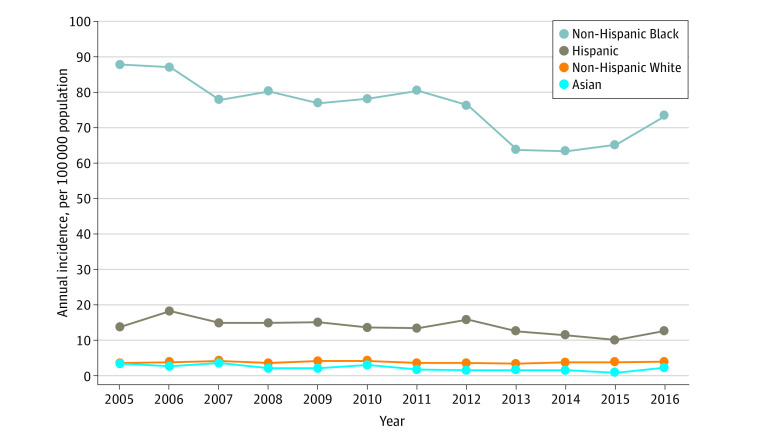
Annual Incidence of Nonfatal Firearm-Related Injuries in New York From 2005 to 2016 by Race/Ethnicity

The overall annual incidence of nonfatal firearm-related injuries in New York over the study period was 18.4 per 100 000 population. The annual incidence of nonfatal firearm-related injuries was 9 times higher among male individuals than among female individuals (34.9 vs 3. 8 per 100 000 population). Non-Hispanic Black individuals had the highest incidence of nonfatal firearm-related injuries (75.5 per 100 000 population); this incidence was 40 times higher than that among Asian individuals (1.9 per 100 000 population), 21 times higher than that among White individuals (3.6 per 100 000 population), and 6 times higher than that among Hispanic individuals (13.6 per 100 000 population) (Figure 1).

**Figure 2.**
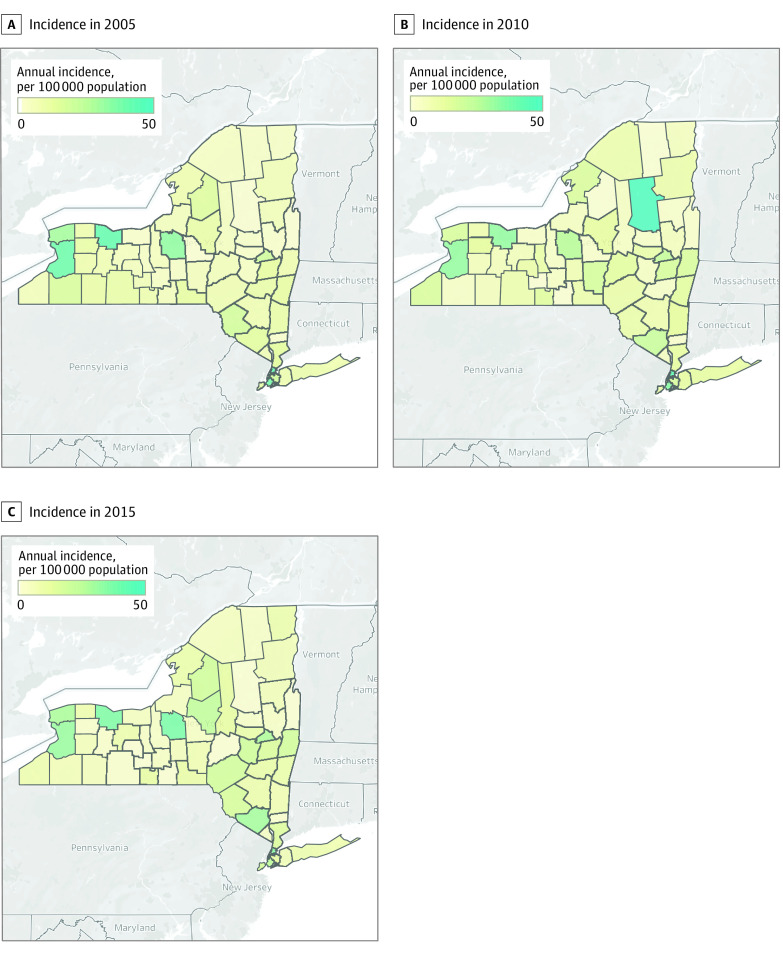
Annual Incidence of Nonfatal Firearm-Related Injuries in New York Counties in 2005, 2010, and 2015 Images are from ©OpenStreetMap contributors. Data are available under the Open Database License (https://www.openstreetmap.org/copyright).

Geographic disparities were observed among counties (Figure 2), with interquartile ranges of 4.1 to 11.8 per 100 000 population in 2005, 3.9 to 14.1 per 100 000 population in 2010, and 3.4 to 15.2 per 100 000 population in 2015. The annual incidence in the state decreased from 20.5 per 100 000 population in 2005 to 18.8 per 100 000 population in 2010 (*P* < .001) and then to 16.8 per 100 000 population in 2015 (*P* < .001). However, the annual incidence increased to 18.8 per 100 000 population in 2016 (*P* < .001).

**Figure 3.**
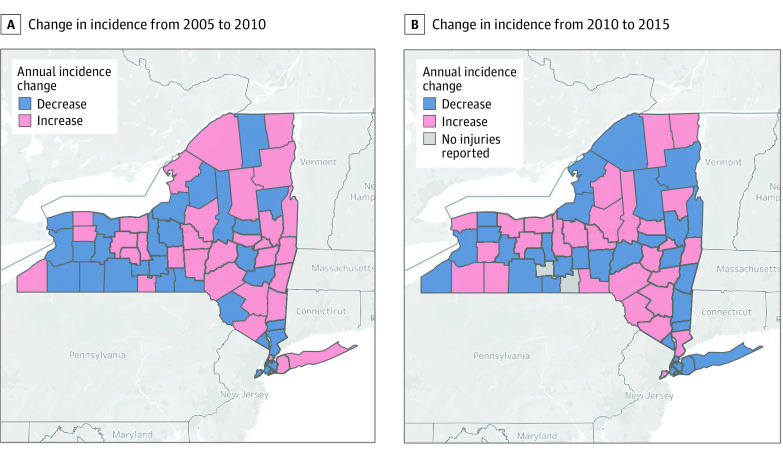
Changes in the Annual Incidence of Nonfatal Firearm-Related Injuries in New York Counties From 2005 to 2010 and From 2010 to 2015 Images are from ©OpenStreetMap contributors. Data are available under the Open Database License (https://www.openstreetmap.org/copyright).

Despite the decreasing overall annual incidence during the study period, the decrease was not consistent among all counties. Between 2005 and 2010 (Figure 3A), 32 (51.6%) counties had increasing annual incidence; between 2010 and 2015 (Figure 3B), 29 counties (46.8%) had increasing incidence. Among the 30 counties with decreases in firearm-related injuries between 2005 and 2010, 19 (63.3%) had an increase in incidence in the later period.

**Figure 4.**
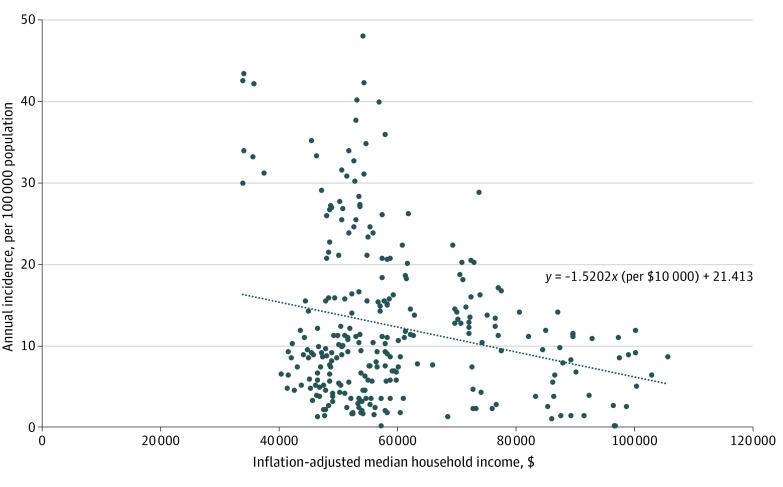
Correlation Between Median Household Income and Annual Incidence of Nonfatal Firearm-Related Injuries at the County Level in New York From 2010 to 2016 Dotted line indicates fitted regression.

The correlation between the annual incidence of nonfatal firearm-related injuries and median household income at the county level from 2010 to 2016 is presented in Figure 4. Counties with lower median household income had a greater annual incidence of nonfatal firearm-related injuries, but the result was only statistically significant before accounting for county-level clustering (unclustered analysis: regression coefficient, −1.520 per $10 000 [95% CI, −2.269 to −0.772]; *P* < .001; after accounting for county-level clustering: regression coefficient, −1.520 per $10 000 [95% CI, −3.149 to 0.108]; *P* = .07). Median household income was not correlated with the change in annual incidence of nonfatal firearm-related injuries between 2010 and 2015 (regression coefficient, 0.034 per $10 000; 95% CI, −1.120 to 1.188; *P* = .95).

## Discussion

This cross-sectional analysis demonstrated the feasibility of using a comprehensive state-level database to assess the annual incidence of nonfatal firearm-related injuries. The annual incidence of nonfatal firearm-related injuries in New York between 2005 and 2016 was 18.4 per 100 000 population, which was approximately 3 times the incidence (5.9 per 100 000 population) of fatal firearm-related injuries during the same period.^15^ Racial/ethnic disparities were substantial, with non-Hispanic Black individuals sustaining the highest incidence of nonfatal injuries. Furthermore, even though the overall incidence of firearm-related injuries in New York decreased significantly during the study period, important geographic disparities were observed.

Nonfatal firearm-related injuries are incompletely recorded at the federal level.^4,6^ The lack of systematic collection and reporting of data on nonfatal firearm-related injuries limits the ability to examine survivors' long-term health and the full impact of firearm-related injuries, including direct and indirect health care costs and other sequelae associated with these injuries.^6^ Surviving firearm-related injuries has been associated with increased risk for chronic pain and other chronic medical conditions, including substance use and mental health disorders, underscoring the need to systematically improve care for this patient population.^16,17^

The use of a comprehensive, statewide database adds to the literature attempting to quantify the scope of nonfatal firearm-related injuries. A previous study^18^ in which nonfatal firearm-related injury rates were reported relied mainly on data sampled from a proportion of emergency department visits that were weighted to estimate national rates. With use of the New York SPARCS, the present study could assess a comprehensive count of injuries for an entire state rather than sampled data, more granular data at the county level, and variation across counties within the same state. Our study showed that existing health care system clinical data can be used to provide more comprehensive information regarding these injuries and suggests that improvement in the infrastructure to facilitate this strategy nationally is needed.

Although the overall state-level incidence of firearm-related injuries decreased over time, this overall decrease was not distributed uniformly across counties. This variation likely reflects the complex ways that socioeconomic and other factors at the neighborhood level may be associated with nonfatal firearm-related injuries. The literature on the geographic disparities in the incidence of firearm-related injuries over time is limited.^19^ More research is needed to evaluate the association between temporospatial changes in sociodemographic factors and the incidence of firearm-related violence.

Our analysis also suggests that the racial disparities in the incidence of firearm homicides^20,21^ may be similar to those in the incidence of nonfatal firearm-related injuries. In the present study, non-Hispanic Black individuals had the highest incidence of nonfatal firearm-related injuries, sustaining nonfatal firearm-related injuries at a rate 40 times higher than that among Asian individuals, 21 times higher than that among White individuals, and 6 times higher than that among Hispanic individuals. Previous literature has demonstrated factors associated with firearm-related injuries, including income inequality,^22,23^ lack of trust in institutions,^24^ lack of government spending on education and social and public services,^25^ barriers to social mobility,^26^ and racial segregation.^27,28^ Racial segregation and lack of government spending on social safety nets and education systems can affect upward social mobility and is associated with interpersonal violence.^26^ We found that men had a 9 times higher annual incidence of nonfatal firearm-related injuries than women. The finding is consistent with the results reported by Gani et al,^29^ who used the Nationwide Emergency Department Sample data in the US from 2006 to 2014.

Better understanding of the mechanisms underlying the decreasing incidence of nonfatal firearm-related injuries in New York, which decreased from 20.5 per 100 000 population in 2005 to 16.8 per 100 000 population in 2015 (before increasing again in 2016), is an important area for continued study. The regulatory approach to firearms in New York may be 1 factor. Several studies have described an association between strict gun laws and a lower incidence of firearm-related fatalities.^30,31,32,33^ The overall decrease in nonfatal firearm-related injuries in New York coincided with the enactment of strict laws in New York regulating the purchase and possession of certain firearms during this same time frame. For example, between 1992 and 2016, the New York state legislature enacted 40 new firearm provisions, including the Secure Ammunition and Firearms Enforcement Act of 2013, New York’s omnibus gun control law^34^; the same number of firearm injury prevention laws passed in Hawaii and was only surpassed by Massachusetts (45), California (46), Maryland (47), and Connecticut (62).^35^ Given challenges with confounding owing to states with fewer firearms often being those that pass the most restrictive gun laws, the causal relationship between specific laws and fatal and nonfatal firearm-related injuries is an important area for future study.

### Limitations

This study has limitations. First, the calculations of nonfatal firearm-related injuries presented in this study contained uncertainty because some individuals who sustained a nonfatal firearm-related injury may not have sought medical care. Although prior analyses suggest that this number is less than 10%,^36^ it is difficult to quantify precisely. Second, the data were limited to New York and did not capture people from New York who were hospitalized in another state. It is unknown whether the trends found in New York are generalizable across other states and regions, again suggesting the importance of having a national data infrastructure to better evaluate nonfatal firearm-related injuries. Third, owing to SPARCS data availability, only data through 2016 were available for this analysis. The absence of real-time reporting of these data, which would be most useful for directing interventions, suggests the need to increase investment in data reporting systems substantially. Fourth, the SPARCS diagnosis codes transitioned from *ICD-9-CM* to *ICD-10-CM* in October 2015. The transition may have led to inconsistencies in the eligibility and identification of those included in this study.^37^

## Conclusions

In this cross-sectional study, the overall annual incidence of nonfatal firearm-related injuries in New York over the study period was 18.4 per 100 000 population. For each person who died of a firearm-related injury, 3 individuals survived.^15^ Complete nonfatal injury data may be useful for policy makers, hospital systems, community organizers, and public health officials when they consider resource allocation for trauma systems and injury prevention programs. Investment in improved reporting of nonfatal injuries appears to be needed to inform a national effort to improve care for survivors of these injuries and to gain a better understanding of the totality of firearm-related violence in the US.

## Notes

Supplement. **eTable.** International Classification of Diseases (ICD), 9th and 10th edition, clinical modification (CM) code used to define firearm-related injuries

## References

[1] Centers for Disease Control and Prevention, National Center for Health Statistics. All injuries. 2017. Accessed June 11, 2020. https://www.cdc.gov/nchs/fastats/injury.htm

[2] Centers for Disease Control and Prevention, National Center for Health Statistics. CDC WONDER: compressed mortality, 1999-2016. 2018. Accessed July 30, 2020. https://wonder.cdc.gov/cmf-icd10.html

[3] Stark DE, Shah NH. Funding and publication of research on gun violence and other leading causes of death. JAMA. 2017;317(1):84-85. doi:10.1001/jama.2016.16215 28030692

[4] Everytown Research & Policy. A more complete picture: the contours of gun injury in the United States. Accessed December 12, 2020. https://everytownresearch.org/report/nonfatals-in-the-us/

[5] Centers for Disease Control and Prevention. Injury prevention and control: WISQARS—Web-based Injury Statistics Query and Reporting System, nonfatal injury data, 2003. Accessed June 2, 2021. https://www.cdc.gov/injury/wisqars/nonfatal.html

[6] Campbell S, Nass D, Nguyen M. The CDC is publishing unreliable data on gun injuries: people are using it anyway. FiveThirtyEight. 2018. Accessed December 12, 2020. https://fivethirtyeight.com/features/the-cdc-is-publishing-unreliable-data-on-gun-injuries-people-are-using-it-anyway/

[7] New York State Department of Health. Statewide Planning and Research Cooperative System (SPARCS). Accessed April 28, 2021. https://www.health.ny.gov/statistics/sparcs/

[8] Parikh K, Silver A, Patel SJ, Iqbal SF, Goyal M. Pediatric firearm-related injuries in the United States. Hosp Pediatr. 2017;7(6):303-312. doi:10.1542/hpeds.2016-0146 28536190

[9] Harrison J. ICD-coding of firearm injuries. Centers for Disease Control and Prevention, National Center for Health Statistics. 2018. Accessed March 29, 2021. https://www.cdc.gov/nchs/injury/ice/amsterdam1998/amsterdam1998_guncodes.htm

[10] Centers for Disease Control and Prevention. Injury prevention and control: matrix of E-code groupings. 2018. Accessed March 29, 2021. https://www.cdc.gov/injury/wisqars/ecode_matrix.html

[11] US Census Bureau. American Community Survey: demographic and housing estimates—2005-2015 American Community Survey 1-year estimates. Accessed April 15, 2021. https://www.census.gov/programs-surveys/acs/data.html

[12] US Census Bureau. American Community Survey: median income in the past 12 months—2010 and 2015 American Community Survey 1-year estimates. Accessed April 15, 2021. https://www.census.gov/programs-surveys/acs/data.html

[13] US Census Bureau. American Community Survey: median income in the past 12 months, 2005. Accessed April 15, 2021. https://www2.census.gov/programs-surveys/acs/summary_file/2005/documentation//

[14] Federal Reserve Bank of Minneapolis. Consumer Price Index, 1913. Accessed April 27, 2021. https://www.minneapolisfed.org/about-us/monetary-policy/inflation-calculator/consumer-price-index-1913-

[15] Centers for Disease Control and Prevention. Injury prevention and control: WISQARS—Web-based Injury Statistics Query and Reporting System. 2003. Accessed April 1, 2021. https://www.cdc.gov/injury/wisqars/index.html

[16] Joseph B, Hanna K, Callcut RA, Coleman JJ, Sakran JV, Neumayer LA. The hidden burden of mental health outcomes following firearm-related injuries. Ann Surg. 2019;270(4):593-601. doi:10.1097/SLA.0000000000003473 31318795

[17] Vella MA, Warshauer A, Tortorello G, . Long-term functional, psychological, emotional, and social outcomes in survivors of firearm injuries. JAMA Surg. 2020;155(1):51-59. doi:10.1001/jamasurg.2019.4533 31746949 PMC6902182

[18] Kaufman EJ, Wiebe DJ, Xiong RA, Morrison CN, Seamon MJ, Delgado MK. Epidemiologic trends in fatal and nonfatal firearm injuries in the US, 2009-2017. JAMA Intern Med. 2021;181(2):237-244. doi:10.1001/jamainternmed.2020.6696 33284327 PMC7851729

[19] Zebib L, Stoler J, Zakrison TL. Geo-demographics of gunshot wound injuries in Miami-Dade county, 2002-2012. BMC Public Health. 2017;17(1):174. doi:10.1186/s12889-017-4086-1 28178967 PMC5299649

[20] Fowler KA, Dahlberg LL, Haileyesus T, Annest JL. Firearm injuries in the United States. Prev Med. 2015;79:5-14. doi:10.1016/j.ypmed.2015.06.002 26116133 PMC4700838

[21] Kalesan B, Adhikarla C, Pressley JC, . The hidden epidemic of firearm injury: increasing firearm injury rates during 2001–2013. Am J Epidemiol. 2017;185(7):546-553. doi:10.1093/aje/kww147 28338922

[22] Kennedy BP, Kawachi I, Prothrow-Stith D, Lochner K, Gupta V. Social capital, income inequality, and firearm violent crime. Soc Sci Med. 1998;47(1):7-17. doi:10.1016/S0277-9536(98)00097-5 9683374

[23] Rowhani-Rahbar A, Quistberg DA, Morgan ER, Hajat A, Rivara FP. Income inequality and firearm homicide in the US: a county-level cohort study. Inj Prev. 2019;25(suppl 1):i25-i30. doi:10.1136/injuryprev-2018-043080 30782593

[24] Woods-Jaeger B, Berkley-Patton J, Piper KN, O’Connor P, Renfro TL, Christensen K. Mitigating negative consequences of community violence exposure: perspectives from African American youth. Health Aff (Millwood). 2019;38(10):1679-1686. doi:10.1377/hlthaff.2019.00607 31589537

[25] Tracy BM, Smith RN, Miller K, . Community distress predicts youth gun violence. J Pediatr Surg. 2019;54(11):2375-2381. doi:10.1016/j.jpedsurg.2019.03.021 31072680

[26] Manduca R, Sampson RJ. Punishing and toxic neighborhood environments independently predict the intergenerational social mobility of black and white children. Proc Natl Acad Sci U S A. 2019;116(16):7772-7777. doi:10.1073/pnas.1820464116 30936309 PMC6475375

[27] Cheon C, Lin Y, Harding DJ, Wang W, Small DS. Neighborhood racial composition and gun homicides. JAMA Netw Open. 2020;3(11):e2027591. doi:10.1001/jamanetworkopen.2020.27591 33252687 PMC7705591

[28] Krivo LJ, Peterson RD, Kuhl DC. Segregation, racial structure, and neighborhood violent crime. AJS. 2009;114(6):1765-1802. doi:10.1086/597285 19852253

[29] Gani F, Sakran JV, Canner JK. Emergency department visits for firearm-related injuries in the United States, 2006–14. Health Aff (Millwood). 2017;36(10):1729-1738. doi:10.1377/hlthaff.2017.0625 28971917

[30] Simonetti JA, Rowhani-Rahbar A, Mills B, Young B, Rivara FP. State firearm legislation and nonfatal firearm injuries. Am J Public Health. 2015;105(8):1703-1709. doi:10.2105/AJPH.2015.302617 26066935 PMC4504301

[31] Reeping PM, Cerdá M, Kalesan B, Wiebe DJ, Galea S, Branas CC. State gun laws, gun ownership, and mass shootings in the US: cross sectional time series. BMJ. 2019;364:l542. doi:10.1136/bmj.l542 30842105 PMC6402045

[32] Fleegler EW, Lee LK, Monuteaux MC, Hemenway D, Mannix R. Firearm legislation and firearm-related fatalities in the United States. JAMA Intern Med. 2013;173(9):732-740. doi:10.1001/jamainternmed.2013.1286 23467753

[33] Santaella-Tenorio J, Cerdá M, Villaveces A, Galea S. What do we know about the association between firearm legislation and firearm-related injuries? Epidemiol Rev. 2016;38(1):140-157. doi:10.1093/epirev/mxv012 26905895 PMC6283012

[34] New York State. NY SAFE Act. Accessed December 26, 2020. https://safeact.ny.gov/

[35] Siegel MB. State firearm laws. Accessed January 13, 2021. https://www.statefirearmlaws.org/state-state-firearm-law-data

[36] May JP, Hemenway D, Hall A. Do criminals go to the hospital when they are shot? Inj Prev. 2002;8(3):236-238. doi:10.1136/ip.8.3.236 12226123 PMC1730897

[37] Heslin KC, Owens PL, Karaca Z, Barrett ML, Moore BJ, Elixhauser A. Trends in opioid-related inpatient stays shifted after the US transitioned to *ICD-10-CM* diagnosis coding in 2015. Med Care. 2017;55(11):918-923. doi:10.1097/MLR.0000000000000805 28930890

