# Supplementary material for: Annual Incidence of Hospitalization for Nonfatal Firearm-Related Injuries in New York From 2005 to 2016
Source: JAMA Netw Open. 2021 Jul 28;4(7):e2115713. doi: 10.1001/jamanetworkopen.2021.15713 (PMC8319760; doi:10.1001/jamanetworkopen.2021.15713)
Supplement: Supplement. — eTable. International Classification of Diseases (ICD), 9th and 10th edition, clinical modification (CM) code used to define firearm-related injuries [file jamanetwopen-e2115713-s001.pdf]

Supplementary Online Content

Hsu YT, Chen YW, Chang DC, et al. Annual incidence of hospitalization for nonfatal firearm-related injuries in New York from 2005 to 2016. *JAMA Netw Open*. 2021;4(7):e2115713. doi:10.1001/jamanetworkopen.2021.15713

eTable. International Classification of Diseases (ICD), 9th and 10th edition, clinical modification (CM) code used to define firearm-related injuries

This supplementary material has been provided by the authors to give readers additional information about their work.

© 2021 Hsu YT et al. *JAMA Network Open*.

eTable 1. International Classification of Diseases (ICD), 9th and 10th edition, clinical modification (CM) codes used to define firearm-related injuries

ICD-10 Codes				
Manner/Intent				
**Accident**	**Intentional self-harm**	**Assault**	**Undetermined intent**	**Legal intervention/ Operation of War**
W32, W33, W34.00, W34.09, W34.10, W34.19	X72, X73, X74.8, X74.9	X93, X94, X95.8, X95.9	Y22, Y23, Y24.8, Y24.9	Y35.00–Y35.03<br>Y35.09<br>Y36.42<br>Y36.43<br>Y36.92<br>Y37.42<br>Y37.43<br>Y37.92<br>Y38.4
**ICD-9 Codes**				
**Manner/Intent**				
**Unintentional**	**Self-inflicted**	**Assault**	**Undetermined**	**Legal intervention**
E922.0-.3,.8, .9	E955.0-.4	E965.0-4, E979.4	E985.0-.4	E970

© 2021 Hsu YT et al. *JAMA Network Open*.
